# Influence of coprecipitation synthesis parameters on the physicochemical properties and biological effects of iron oxide nanoparticles

**DOI:** 10.1039/d5na00632e

**Published:** 2025-10-02

**Authors:** Marco Reindl, Verena Zach, Gerhard Cvirn, Sebastian P. Schwaminger

**Affiliations:** a Medical University of Graz, Otto Loewi Research Center, Division of Medicinal Chemistry, Nano Lab Neue Stiftingtalstraße 6 8010 Graz Austria sebastian.schwaminger@medunigraz.at; b Medical University of Graz, Otto Loewi Research Center, Division of Medicinal Chemistry Neue Stiftingtalstraße 6 8010 Graz Austria; c Technical University of Munich, TUM School of Engineering and Design, Chair of Bioseparation Engineering Boltzmannstr. 15 85748 Garching Germany s.schwaminger@tum.de; d BioTechMed-Graz Mozartgasse 12 8010 Graz Austria

## Abstract

Iron oxide nanoparticles (IONPs) are promising candidates for a variety of biomedical applications thanks to their magnetic properties and biocompatibility. However, optimising their physicochemical and biological behaviour through fine-tuning their synthesis remains challenging. In this study, we systematically investigated the effect of variations in coprecipitation synthesis parameters, including Fe^2+^/Fe^3+^ ratios, NaOH concentration, stirring speed, temperature and dosing rate, on IONP size, cytotoxicity and blood coagulation. Our robust regression model (*R*^2^ = 0.91) revealed that the concentration of Fe^2+^ and its interaction with the other synthesis factors had a strong influence on particle size, which ranged from 3 to 16 nm while maintaining the magnetite phase. All synthesised nanoparticles demonstrated excellent biocompatibility, with no evidence of cytotoxicity observed. Notably, all IONPs induced a significant reduction in coagulation time (CT), indicating a procoagulant effect modulated by synthesis conditions: higher Fe^3+^ values prolonged CT, whereas increased Fe^2+^ values accelerated clotting. Particle size predominantly influenced clot formation time (CFT) and clot firmness without compromising final clot stability, suggesting nuanced modulation of haemostasis. These findings emphasise the critical role of nanoparticle surface chemistry and synthesis control in tailoring IONP properties for biomedical applications.

## Abstract 2

Investigation of coprecipitation parameters on IONP physicochemistry and bioactivity.

## Graphical abstract

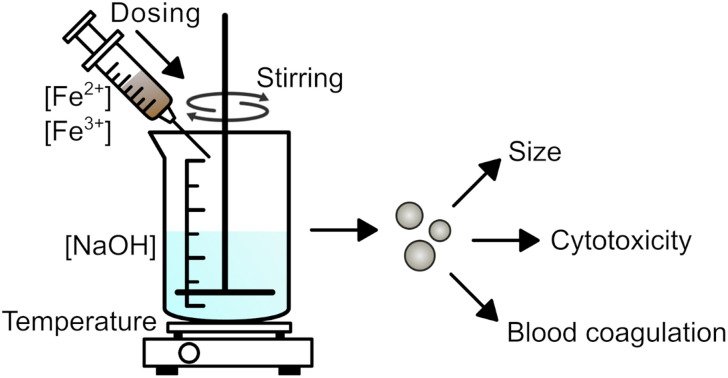

## Introduction

Nanomedicine is evolving rapidly, driven by the continuous development of novel materials that enable innovative strategies for diagnostics, therapeutics, and drug delivery.^1,2^ One prominent example of this progress is iron oxide nanoparticles (IONPs). IONPs are highly valued for their unique properties, such as superparamagnetism, biocompatibility, and high surface-to-volume ratio.^3^ Therefore, they are widely used in a broad range of biomedical applications. In magnetic particle imaging, they serve as tracers, offering advantages such as linear quantification, positive contrast, absence of radiation, unlimited penetration, and no background interference. These properties make IONPs ideal for applications like cell tracking, and tumour and blood imaging.^4^ In magnetic hyperthermia therapy, IONPs are accumulated in tumour tissue and selectively destroy cancer cells by generating heat in response to an external magnetic field.^5^ In tissue engineering, magnetic-driven strategies allow non-contact manipulation of engineered living modules, opening new possibilities for skeletal tissue fabrication.^6^

In targeted drug delivery, IONPs can either act directly as drug carriers^7^ or functionalised with coatings, such as silica,^8^ lipids,^9^ or polymers,^10^ to tailor their surface for specific applications.

The synthesis method used to produce IONPs plays a critical role in determining their properties and can significantly affect nanoparticle morphology, which in turn influences important characteristics, such as aggregation, magnetisation, and interactions with the surrounding environment.^11–13^ These factors are essential in determining the suitability of IONPs for various biomedical applications. Among the most widely used methods for synthesising IONPs are thermal decomposition,^14^ hydrothermal synthesis,^15^ polyol synthesis,^16^ sol–gel methods,^17^ and coprecipitation,^3^ each offering different levels of control over particle size, shape, and crystallinity.

Thermal decomposition provides a good control over particle size and crystallinity, although it can sometimes lead to structural defects and polycrystallinity, which may reduce the magnetic properties of the nanoparticles.^14^ While high temperatures can favour the formation of crystalline structures, they also create a risk of nonmagnetic phases, such as wüstite, in oxygen-deficient conditions. However, the application of an oxidation step can help mitigate this.^18^ Despite its potential drawbacks, thermal decomposition remains a viable method for producing nanoparticles with controlled size and crystallinity. Hydrothermal synthesis yields highly crystalline particles but with inconsistent sizes. It also requires long reaction times and high-pressure conditions, making it less cost-effective than coprecipitation.^3,19^ Despite its ability to produce high-quality nanoparticles, the inconsistencies in size and high operational costs remain significant limitations. Another synthesis approach is polyol synthesis. This method produces biocompatible IONPs with enhanced heating ability and colloidal stability in a short reaction time.^16^ A disadvantage of this synthesis method is that they require additional treatment or a post-synthesis ligand exchange to achieve water dispersibility, a process that is both time-consuming and complex.^20^ The sol–gel method offers precise control over size, shape, and crystallinity.^19^ However, it is time-consuming, prone to aggregation and requires careful sol preparation and post-treatment.^17,21^ In contrast, coprecipitation, especially the Massart process, is a simple, fast, and cost-effective technique.^3,22^ It produces 4 to 16 nm superparamagnetic nanoparticles with high magnetisation, making them ideal for biomedical applications, including drug delivery, imaging, and therapy.^7,10,23^

In a previous study, we optimised the synthesis of IONPs *via* the coprecipitation method, exploring the impact of the concentration of sodium hydroxide (NaOH), ferrous chloride and ferric chloride, temperature, stirring speed, and dosing rate on the particle size and growth. Using small-angle X-ray scattering (SAXS), it was observed that higher temperatures led to a 50% increase in particle size, while the stirring speed and NaOH concentration also influenced nucleation and aggregation.^24^ These findings highlight the importance of understanding how synthesis parameters influence various outcome characteristics to achieve more reproducible results, ensuring low synthesis-to-synthesis variation in important characteristics such as particle size, iron oxide phase, and biocompatibility. Despite the broad range of studies on the synthesis and application of IONPs, there remains a gap in understanding how variations in synthesis conditions affect their biological behaviour, particularly regarding the interactions between IONPs and blood.^25,26^ The role of IONPs in the bloodstream, such as their influence on blood clotting is of significant concern when considering their *in vivo* use.^27^ IONPs can interact with blood proteins and cells, potentially altering coagulation pathways, which could lead to serious complications such as thrombosis or haemorrhage.^26,28^ This is especially important for applications like drug delivery and cancer treatment, where nanoparticles may directly circulate in the bloodstream.

In this study, we systematically investigate how different coprecipitation conditions influence the iron oxide phase, size, cytotoxicity, and blood coagulation properties of IONPs. By studying the impact of synthesis conditions on these parameters, this research aims to contribute valuable insights into the design of safe IONPs for clinical applications, ensuring their reproducibility, biocompatibility, and minimised risks in therapeutic and diagnostic procedures.

## Experimental

### Materials

Iron(ii)chloride tetrahydrate (98%), iron(iii)chloride anhydrous (97%), and sodium hydroxide pellets (≥97%), were purchased from Sigma-Aldrich Handels GmbH (Vienna, Austria). Calcium chloride (≥94%) was purchased from Carl Roth GmbH + Co. KG (Karlsruhe, Germany). Dulbecco's Modified Eagle Medium (DMEM, 4.5 g L^−1^ glucose, 2 mM l-glutamine), foetal bovine serum (FBS), penicillin–streptomycin (10.000 U mL^−1^), and XTT assay kit were purchased from Thermo Fisher Scientific GmbH (Vienna, Austria). Normocin and HEK-Blue Selection were purchased from InvivoGen SAS (Toulouse, France). TF thromboplastin (Innovin®) was obtained from Dade Behring Marburg GmbH (Marburg, Germany).

### Synthesis of IONPs

Superparamagnetic IONPs were prepared by coprecipitation following the Massart process.^29^ An overview of the amounts of substances and synthesis conditions is provided in Table 1. A comprehensive list of all amounts of substances and synthesis conditions is given in Table S1.

**Table 1:** Investigated factors and their corresponding levels used in the experimental design. Factors are given at three levels: −1, 0, and 1, representing low, medium, and high conditions, respectively

Factor	−1	0	1
[Iron(ii)chloride] (mmol)	1.76	—	3.52
[Iron(iii)chloride] (mmol)	3.20	—	6.39
Sodium hydroxide (mmol)	18	—	38
Temperature (°C)	30	55	80
Stirring speed (rpm)	0	500	1000
Dosing rate (s)	10	30	60

For the synthesis, the respective amount of NaOH was dissolved in 15 mL ultrapure water and adjusted to the assigned temperature. Ferrous chloride and ferric chloride were dissolved in 6 mL ultrapure water. Under continuous mechanical stirring, the iron salts were added to the basic solution at a pre-defined dosing rate. The reaction was allowed to continue for 30 minutes after the complete addition of the iron salts. The synthesised nanoparticles (IONPs) were washed 15 times by magnetic decantation with ultrapure water. IONPs were resuspended in ultrapure water and the mass concentration determined by gravimetry. The particles were stored at RT for further analysis.

### Attenuated total reflectance Fourier-transform infrared spectroscopy (ATR-FTIR)

Iron oxide phase was assessed by ATR-FTIR. The respective nanoparticle suspension was placed on the ATR crystal and the liquid was evaporated. The data were recorded (4 scans) using a UATR-FTIR (Spectrum Two, PerkinElmer, Inc.) equipped with a diamond ATR crystal and DTGS detector at room temperature. The analysis of the iron oxide phase was performed in the spectroscopy software Spectragryph.^30^

### Transmission electron microscopy

Morphology and size of the IONPs was assessed by transmission electron microscopy (Tecnai G20, FEI Company), operated at a voltage of 120 kV. The nanoparticles were diluted to a concentration of 10 mg L^−1^ with ultrapure water and redispersed using an ultrasonic processor (Model 120 Sonic Dismembrator, Fisherbrand). The suspension was placed on a carbon-coated copper grid (200 mesh, Ted Pella, Inc.), which had been glow discharged using a PELCO easiGlow device (Ted Pella, Inc.). Images were obtained using a BM-Ultrascan 1000P CCD camera (Gatan, Inc.). The particle size and morphology was analysed using Fiji.^31^ The average size was calculated from at least 96 measurements after outlier removal using the interquartile range method.

### Cytotoxicity assay in mammalian cells

Cytotoxicity of IONPs was verified with an XTT cell proliferation assay (CyQUANT XTT Cell Viability Assay, Invitrogen) in HEK-Blue TLR4 and 3T3-L1 mouse fibroblasts. The assay was conducted following the instructions outlined in the manual. HEK cells were seeded at a density of 6000 cells in 100 μL of medium per well, while 3T3 cells were seeded at a density of 1000 cells in 100 μL of medium per well. For the cultivation of 3T3 and HEK-Blue TLR4 cells, DMEM was supplemented with 10% (v/v) heat inactivated FBS and1% penicillin–streptomycin (100 μg mL^−1^). For HEK-Blue TLR4 cells, the medium was additionally supplemented with 1 mL normocin (100 μg mL^−1^) and 2 mL HEK-Blue Selection per 500 mL of medium. Both cell types were plated onto a 96-well plate. The cells were incubated in the respective growth medium at 37 °C and 5% CO_2_ for 48 hours to reach a confluence close to 90%. 50 μL of the reconstituted XTT mixture was added to the cells and mixed well before incubation for 4 hours at 37 °C protected from light. The absorbance was measured at 450 nm and 660 nm with a UV-Vis spectrophotometer (PowerWave Select X, Bio-Tek Instruments, Inc.).

### Rotational thromboelastometry (ROTEM)

Measurements were performed on citrated whole blood (WB) samples. The clot formation process was monitored using the thromboelastometry coagulation analyzer (ROTEM®05, Matel Medizintechnik, Graz, Austria). For the experiments WB samples (360 μL) were incubated with 40 μL of sample solution (containing physiological sodium chloride or nanoparticle suspensions, 80 μg mL^−1^ final concentration) for 30 min with gentle shaking at 37 °C. Clot formation was initiated by the addition of 40 μL “trigger solution” [0.35 pmol L^−1^ recombinant human tissue factor (TF) and 3 mmol L^−1^ calcium chloride] to 300 μL of citrated WB containing the IONPs. The TF stock solution was prepared by dissolving lyophilised TF in 4 mL ultrapure water, followed by a 1 : 250 dilution in 0.9% sodium chloride. This method has been previously described in detail by Sørensen *et al.*^32^ Following laboratory parameters were obtained using ROTEM: Coagulation time (CT), which indicates the time from the addition of the “trigger solution” to the initiation of fibrin formation; clot formation time (CFT), representing the time required to reach a clot amplitude of 20 mm; maximum clot firmness (MCF), reflecting clot stability; and the alpha angle, which describes the rate of fibrin build-up and cross-linking.

### Zeta potential

IONPs were diluted with ultrapure water to a concentration of 1 g L^−1^ and equilibrated to pH 7.0 (±0.05) with either 0.01 M HCl or 0.01 M NaOH. Afterwards, the particles were diluted with ultrapure water to a final concentration of 50 mg L^−1^ and the pH re-verified. The particles were sonicated in an ultrasonic processor (Model 120 Sonic Dismembrator, Fisherbrand) and zeta potential was subsequently analysed 25 °C using a Zetasizer Nano ZS (Malvern Panalytical, Ltd.).

### Data analysis and visualisation

The data was analysed and visualised in Python 3.12.0. For data analysis, SciPy^33^ and statsmodels^34^ packages were used. All data points were included in a RSM model to identify significant parameters influencing particle diameter (measured from transmission electron micrographs) and coagulation parameters, including CT, CFT, MCF, and *α*. The functional form of the RSM model is given by:
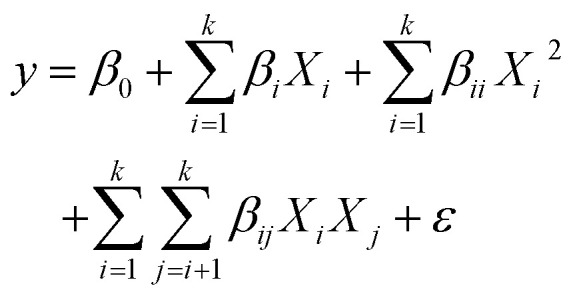
where *X*_*i*_ are the independent variables (Fe^2+^, Fe^3+^, NaOH, temperature, stirring, dosing), *β*_*i*_ are the coefficients for linear terms, *β*_*ii*_ are coefficients for quadratic terms, *β*_*ij*_ are the coefficients for interaction terms, and *ε* is the error term. Outliers were identified using Cook's distance, with the threshold set at 4/number of observations. The final model was then used to summarise the significant parameters. To assess the relationships between particle diameter and coagulation parameters, both Pearson and Spearman correlation analyses were performed. Pearson correlation was used to determine the strength and direction of the linear association between particle diameter and each coagulation parameter. Spearman correlation was included to assess potential non-linear relationships and to provide a robust measure of association that is less sensitive to outliers compared to Pearson correlation. Data visualization was performed with the Matplotlib^35^ package.

## Results and discussion

### Effect on the size of IONPs synthesised *via* varying coprecipitation parameters

For the synthesis, an inverse coprecipitation method was chosen. Thus, the iron salts were gradually added to a pre-heated NaOH solution. This method was selected to promote rapid nucleation under strongly alkaline conditions. It was adapted from the widely used Massart method^29^ and is known to facilitate the synthesis of superparamagnetic IONPs with narrow size distributions.^7,10^ Compared to the conventional approach, the inverse method provides a more uniformly alkaline environment during the early stages of nucleation.

**Fig. 1:**
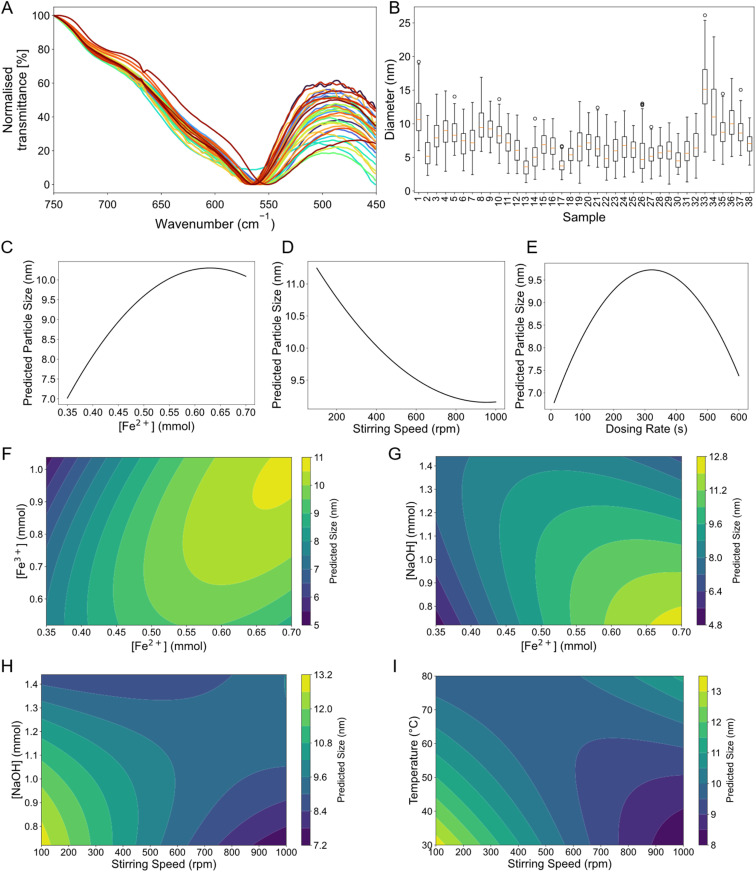
Characterization of IONPs synthesized under different conditions and effect of investigated factors on particle diameter. (A) ATR-FTIR showing the normalized transmittance [%] between 450 and 750 cm^−1^. (B) Particle diameter of the respective samples as determined by transmission electron microscopy (*n* = 96). (C) Relation between predicted particle size and Fe^2+^ concentration, (D) stirring speed, and (E) dosing rate. Relation between (F) Fe^2+^ and Fe^3+^ concentrations, (G) Fe^2+^ and NaOH concentrations, (H) NaOH concentration and stirring speed, and (I) temperature and stirring speed on particle size while other parameters were kept constant at average value.

All samples exhibited the characteristic Fe–O vibrational mode at 565 cm^−1^ (Fig. 1A), consistent with the spinel structure of magnetite-based nanoparticles.^36^ The position of the Fe–O peak indicates that the IONPs mainly consist of magnetite, as maghemite (630–660 cm^−1^) and haematite (540 cm^−1^) typically show Fe–O peaks at differing wavenumbers.^37^ Interestingly, this result suggests that varying synthesis conditions did not influence the iron oxide phase of the nanoparticles indicating the robustness of the particle synthesis by coprecipitation in terms of iron oxide phase.

Subsequent analysis of the morphology and size showed a more diverse picture when comparing to ATR-FTIR results. The morphology of the IONPs was assessed by transmission electron microscopy (TEM). The majority of the IONPs showed a morphology which can be described as mostly round to ellipsoid (1, 2, 4, 6, 9, 10, 16, 18–24, 28, 30–34, 36–38), two samples showed mostly round to ellipsoid with a few cubic particles (11, 12), a mix of round to ellipsoid and cubic particles was observed in several samples (3, 5, 7, 8, 25–27, 29, 35), while very irregularly shaped particles were only found in three samples (13, 14, 17) (Fig. S1A–AL). The average diameter of the particles ranged between 3.7 and 16.5 nm (Fig. 1B, Table S2) indicating a moderate variability upon varying synthesis factors. Still, all the particles fall in an acceptable range when comparing it to previously reported size distributions of IONPs synthesised *via* coprecipitation usually ranging from 4 to 16 nm.^7,10,38^

To better understand the influence of synthesis parameters on particle size, we applied ordinary least squares (OLS) regression analysis within the framework of response surface methodology (RSM). The goal of this model was to predict the average particle size of the IONPs as a function of key experimental factors. The analysis identified several statistically significant parameters and interactions (Tables 2 and S3), including the concentrations of Fe^2+^, the Fe^2+^/Fe^3+^ ratio, NaOH concentration, stirring speed, and temperature. Notably, interactions between Fe^2+^ and Fe^3+^, Fe^2+^ and NaOH, NaOH and stirring speed, and temperature and stirring speed also had significant effects. The fitted regression model explained a large portion of the variance in particle size, with an *R*^2^ of 0.95, indicating excellent agreement with the experimental data. The adjusted *R*^2^ of 0.74 confirms that the model remains robust without overfitting, supporting the reliability of the identified trends.

**Table 2:** Investigated synthesis factors which significantly affecting the diameter of the synthesised IONP. **2 indicates quadratic effects. Full table in SI

Factor	Coefficient	Std. error	*t*-Statistic	*p*-Value
Fe^2+^	1.72	0.443	3.88	0.006
Stirring speed	−1.17	0.437	−2.681	0.031
*I* (dosing rate**2)	−2.64	0.864	−3.053	0.019
Fe^2+^ : Fe^3+^	1.02	0.409	2.501	0.041
Fe^2+^ : NaOH	−1.94	0.51	−3.787	0.007
NaOH : stirring speed	1.65	0.566	2.923	0.022
T : stirring speed	1.66	0.537	3.09	0.018

The RSM model revealed that Fe^2+^ concentration has a statistically significant linear effect on particle size (Table 2), with a coefficient (*β*) of 1.72 (*p* = 0.006). This indicates that, holding other variables constant, each 1 mM increase in Fe^2+^ concentration results in an average increase of 1.72 nm in particle diameter (Fig. 1C). However, while the quadratic effect of Fe^2+^ concentration on particle size was not statistically significant (*β* = −1.29, *p* = 0.061), it approaches significance, suggesting also a potential nonlinear relationship, as the relation in Fig. 1C already implicates. This trend aligns with classical nucleation theory, where higher precursor concentrations suppress nucleation and favour particle growth.^39^ The presence of excess Fe^2+^ ions in solution provides a continuous source for crystal enlargement, leading to larger nanoparticles. Similar trends have been reported in iron oxide and ferrite nanoparticle synthesis, where higher Fe^2+^ concentrations correlate with enhanced particle growth due to reduced nucleation rates and prolonged ion availability.^29,40^ Beyond this, increased Fe^2+^ levels have also been linked to under stoichiometric conditions which have the potential to affect particle size through altered oxidation dynamics.^41,42^

However, this deviation from a purely linear trend implies that the growth process cannot be described by a simple linear model. As Fe^2+^ concentration increases, the initial increase in particle size is due to the enhanced availability of Fe^2+^ ions, which favour growth.^39^ Beyond a certain concentration, the rapid consumption of Fe^2+^ ions may lead to increased nucleation and the formation of a greater number of smaller particles. This shift could be attributed to Fe^2+^ depletion in solution, restricting further growth and favouring nucleation of multiple small nanoparticles instead of fewer larger ones. Therefore, the observed quadratic effect suggests that there is an optimal concentration of Fe^2+^, where the dominant process shifts from particle growth to nucleation. At concentrations below this threshold, particle growth dominates, leading to larger nanoparticles. Above this threshold, nucleation becomes more prevalent, resulting in smaller particles. This phenomenon has been previously observed in IONP synthesis, where maintaining the Fe^3+^/Fe^2+^ ratio is challenging due to Fe^2+^ oxidation, further influencing the particle size distribution.^43,44^

Moreover, phase changes at high Fe^2+^ concentrations could further impact particle morphology, as Fe^2+^-rich conditions may promote partial oxidation or transition to non-magnetite phases, predominantly maghemite and haematite.^45^ Magnetite typically forms under reducing conditions with higher Fe^2+^ concentrations, whereas more oxidising environments or limited Fe^2+^ availability tend to favour the formation of maghemite or haematite.^46^ Interestingly, our ATR-FIT data suggests that the predominant phase in the analysed IONPs, irrespective of the Fe^2+^ concentration used for their synthesis, is magnetite (Fig. 1A).

Stirring speed had a statistically significant negative linear effect on particle diameter in the response surface model (*β* = −1.17, *p* = 0.031, Table 2), indicating that, on average, each 100 rpm increase in stirring speed reduces particle size by approximately 1.17 nm (Fig. 1D). This effect can be attributed to enhanced micro-mixing, which improves the homogeneity of Fe^2+^, Fe^3+^, and OH^−^ ion distribution, thereby promoting rapid and uniform nucleation.^24,47^ At lower stirring speeds, diffusion limitations result in localized supersaturation, favouring fewer but larger nuclei that grow into larger particles.^24,47^ Additionally, slow mixing reduces the shear force acting on particles, increasing the likelihood of aggregation and polydispersity.^48^ Equally a higher stirring speed increases the oxygen input in the reaction,^49^ accelerating Fe^2+^ oxidation to Fe^3+^.^50^ This potentially results in higher nucleation rates, as Fe^3+^ is the main driver of iron oxide precipitation, leading to smaller particle sizes due to reduced time for crystal growth.^51^ Moreover, rapid oxidation shifts the Fe^2+^/Fe^3+^ ratio, promoting phase transitions from magnetite to maghemite. Conversely, the ATR-FTIR data do not suggest a shift towards maghemite indicating only a minor effect on the iron oxide phase in our experimental setup (Fig. 1A).

The squared term of the dosing rate shows a coefficient of −4.52 (*p* = 0.039), indicating a decrease in particle size with an increase in the speed of iron salt addition (Fig. 1E and Table 2). In the coprecipitation process, the dosing rate directly influences the nucleation and growth phases of nanoparticle formation.^24^ At higher dosing rates, the rapid introduction of iron salts results in faster nucleation, creating a greater number of smaller nuclei. This increased nucleation rate limits the growth of individual particles, ultimately leading to smaller nanoparticles. Additionally, a higher dosing rate accelerates supersaturation,^52^ which further promotes nucleation over growth, restricting the size of the particles. However, the quadratic nature of the relationship suggests that beyond a certain dosing rate, the reduction in particle size levels off, potentially due to the saturation of available ions and the onset of particle agglomeration.

In the RSM, the ratio between Fe^2+^ and Fe^3+^ was also found to have a significant positive effect on the particle size with a coefficient of 1.02 (*p* = 0.041, Fig. 1F and Table 2). Thus, increasing the Fe^2+^/Fe^3+^ ratio by 1 leads to an increase of approximately 1.02 nm in particle size, holding all other variables constant. Potential explanations for this positive effect are the influence of the Fe^2+^/Fe^3+^ ratio on nucleation and growth dynamics during iron oxide formation. A higher Fe^2+^ concentration is known to reduce the nucleation rate while enhancing particle growth, leading to the formation of larger particles.^53^ Thermodynamic and kinetic considerations further suggest that a higher Fe^2+^/Fe^3+^ ratio can modify the activation energy for nucleation and dissolution-reprecipitation mechanisms, thereby favouring fewer but larger particles.^54,55^

Moreover, the ratio between Fe^2+^ and NaOH exhibits a negative coefficient of −1.94 (*p* = 0.007) on the particle size (Fig. 1G and Table 2). Specifically, an increase of 1 unit in the Fe^2+^/NaOH ratio corresponds to an average decrease of 1.94 nm in particle size, assuming other factors remain constant. The observed negative correlation suggests that increasing NaOH concentration leads to smaller particles under well-mixed conditions. This can be attributed to the role of hydroxide ions in the nucleation and growth process of iron oxides.^56^ Higher NaOH concentrations promote rapid supersaturation, increasing the nucleation rate while limiting subsequent particle growth, resulting in smaller particles.^24,39^ Additionally, a higher concentration of hydroxide ions can accelerate Fe^2+^ hydrolysis and precipitation, potentially leading to a finer distribution of nucleation sites.^45^ Moreover, high hydroxide levels can enhance electrostatic stabilisation of colloidal particles, preventing aggregation and Ostwald ripening.^24,57^

In the RSM, the ratio between NaOH and stirring speed showed a positive effect on particle size with a coefficient of 1.65 (*p* = 0.022, Table 2) suggesting that increased NaOH concentration, relative to stirring intensity, promotes particle growth (Fig. 1H). Holding other factors constant, a one-unit rise in the NaOH-to-stirring speed ratio leads to an average increase of 1.65 nm in particle size. This effect can be explained by the interplay between precipitation kinetics and hydrodynamic conditions during synthesis. While higher NaOH concentrations generally lead to smaller particles by promoting nucleation,^24,39^ their effect is modulated by stirring intensity. The positive effect of NaOH and stirring speed indicates that if the stirring speed is not increased proportionally, it may lead to uneven mixing of the suspension. These conditions can favour particle aggregation or uncontrolled growth, ultimately leading to larger particles.^24,47^ As already discussed earlier, under such lower turbulence conditions, shear-induced fragmentation of growing clusters is also minimized, allowing for enhanced particle growth *via* Ostwald ripening.^48^ Thus, although NaOH alone favours nucleation, its interaction with stirring dynamics determines whether nucleation or growth dominates the process. In addition, the RSM predicts that increasing the temperature-to-stirring speed ratio by one unit results in an average particle size increase of 1.66 nm (*β* = 1.66, *p* = 0.018; Table 2), indicating that elevated temperatures are more effective in promoting particle growth under low stirring conditions (Fig. 1I). As already discussed, a lower stirring rate leads to an increase in particle size due to limited diffusion^24,47^ and reduced shear force.^48^ Temperature influences both the nucleation rate and growth kinetics in iron oxide formation.^47,53^ Higher temperatures generally enhance atomic diffusion and solubility, leading to faster grain growth and coarsening *via* Ostwald ripening.^24^ Thus, the observed effect suggests that under conditions where temperature is elevated but stirring intensity is relatively lower, growth mechanisms dominate over nucleation, leading to larger particle sizes, as expected. Interestingly, a combination of high temperature and high stirring speed can increase the particle size, diminishing the effect of thorough stirring.

Taken together, these results provide important insights into the control over particle size for IONPs synthesised *via* coprecipitation. Fe^2+^ concentration, stirring speed, dosing rate, Fe^2+^/Fe^3+^ and Fe^2+^/NaOH ratios, NaOH and stirring speed interactions, and temperature-stirring speed dynamics, significantly influenced IONP size while maintaining the magnetite phase (Fig. 1A). Higher Fe^2+^ concentrations promote growth, whereas increased stirring speeds reduce particle size by enhancing mixing and Fe^2+^ oxidation.^47,49,50^ The Fe^2+^/Fe^3+^ ratio favours larger particles, while higher NaOH levels drive smaller sizes through rapid nucleation.^24,47,56^ Temperature further supports growth *via* Ostwald ripening.^24,47,53^ Importantly, despite varying the synthesis factors drastically, all particles remained in the magnetite phase (Fig. 1A) as well as in an acceptable size range (Fig. 1B) for IONPs synthesised *via* the Massart process, highlighting the robustness of the coprecipitation method for IONP synthesis. These results demonstrate the possibility to tailor the particle size to meet specific application requirements, which is crucial for ensuring the functionality and effectiveness of the nanoparticles in various fields.^3^

### Cytotoxicity of IONPs synthesised under different conditions

Just as controlling and predicting nanoparticle size is essential for biomedical applications, evaluating their cytotoxicity is equally critical for their safe application in drug delivery and imaging. While IONPs are generally considered biocompatible, factors such as particle surface chemistry and aggregation state can influence their interactions with cells and tissues.^58^ One potential concern is that smaller nanoparticles may generate reactive oxygen species (ROS) due to their high surface area, leading to oxidative stress, DNA damage, or apoptosis.^59^ Additionally, iron homeostasis disruption caused by excess Fe^2+^ or Fe^3+^ ions could interfere with cellular metabolic pathways, potentially leading to ferroptosis.^60,61^ Furthermore, the pH and ionic strength of the biological environment can influence nanoparticle stability and aggregation, altering their cellular uptake and cytotoxic profile.^62,63^ Understanding whether variations in synthesis parameters affect cytotoxicity is essential to ensure reproducibility, safety, and predictability in clinical translation of these IONPs.

**Fig. 2:**
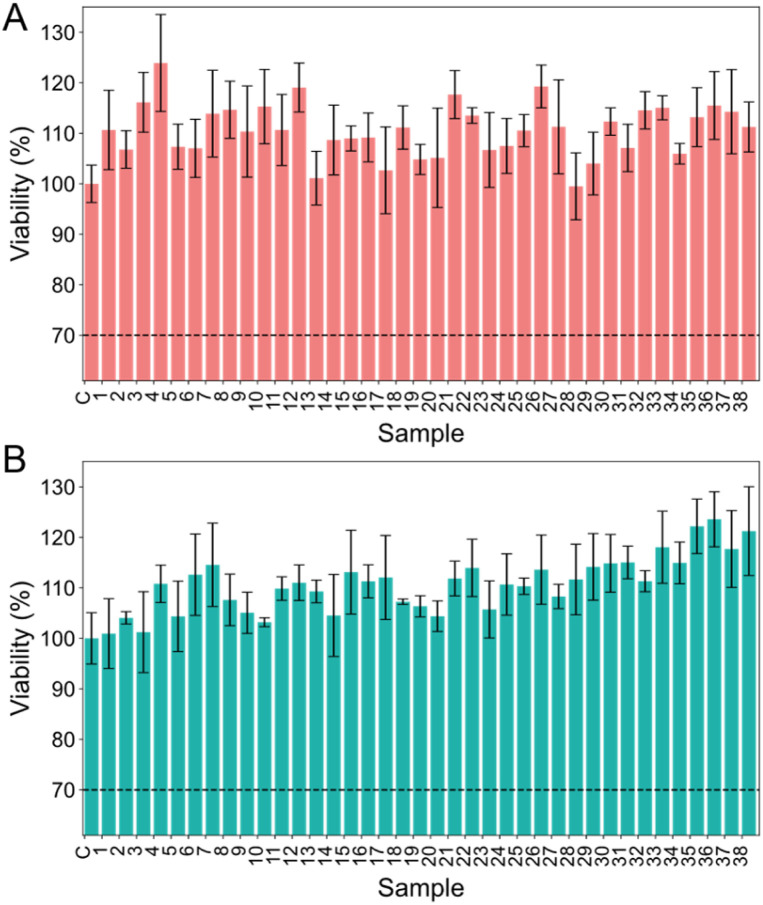
Assessment of cytotoxicity of IONPs synthesised under different conditions in (A) 3T3 mouse embryonic stem cells and (B) HEK cells with a viability threshold at 70% (dashed line). Error bars indicate the standard deviation of the measurement in triplicates.

Statistical analysis revealed no significant correlation between cytotoxicity values and particle sizes, as indicated by both Pearson's and Spearman's correlation coefficients. This suggests that changes in particle size do not meaningfully impact cell viability in our model. Additionally, all synthesized IONPs exhibited cell viability well above 70%, confirming their overall biocompatibility with 3T3 mouse embryonic fibroblasts (Fig. 2A) and HEK cells (Fig. 2B). This suggests that neither variations in Fe^2+^, Fe^3+^, nor NaOH concentrations, nor variations in their ratios had a measurable effect on the cytotoxic effects of the particles. Regardless of the synthesis parameters, the nanoparticles remained non-toxic to both 3T3 mouse embryonic fibroblasts (Fig. 2A) and HEK cells (Fig. 2B). This indicates that the applied synthesis method produces IONPs with consistent biocompatibility, making them suitable for biomedical use.

These findings highlight the robustness of the synthesis method, demonstrating that even drastic variations in precursor concentrations do not compromise cell viability.

### Effect of IONPs synthesised under different conditions on blood coagulation

**Fig. 3:**
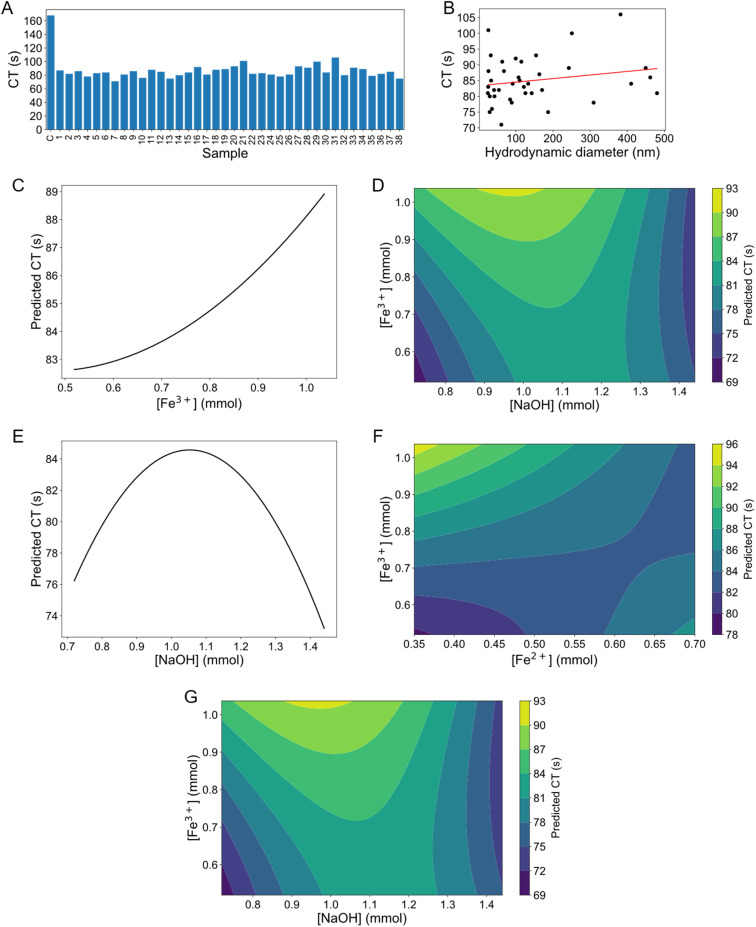
Effect of IONP synthesis parameters and hydrodynamic diameter on coagulation time (CT). (A) Data for CT shown alongside (B) its respective Spearman correlation with IONP hydrodynamic diameter and corresponding regression lines (red). (C) Relation between Fe^3+^ concentration and predicted CT. (D) Effect of ratio between Fe^3+^ and NaOH concentrations on predicted CT. (E) Relation between NaOH concentration and predicted CT. (F) Effect of ratio between Fe^2+^ and Fe^3+^ concentrations on predicted CT. (G) Effect of ratio between Fe^2+^ and NaOH concentrations on predicted CT.

Beyond cytocompatibility, it is important to investigate their possible influence on the human coagulation system to ensure their safe application in clinical settings, particularly with regard to risks such as thrombosis. In the present study, we used rotational thromboelastometry (ROTEM) to evaluate how IONPs synthesised under varying conditions influence the coagulation system. ROTEM is a well-established method for assessing the impact of various effectors on clot formation, offering the advantage of analysing whole blood samples and requiring only small amounts of tissue factor (TF) as an initiator.^32^ This makes it especially valuable for studying coagulation under conditions that closely mimic the *in vivo* environment.^64^ Specifically, we investigated the effects of differently produced IONPs on key ROTEM parameters: coagulation time (CT), clot formation time (CFT), maximum clot firmness (MCF), and the alpha angle. Our experiments clearly indicate that all IONPs investigated herein caused significant prothrombotic effects leading to a CT reduction of 83 s on average (Fig. 3A). Given that CTs were halved under our conditions, it must be assumed that the nanoparticles have a clinically relevant procoagulant effect.

However, ROTEM is a global assay of haemostasis, reflecting the combined activity of platelet function, coagulation proteases, and inhibitors, and the fibrinolytic system. Therefore, the specific mechanisms underlying the procoagulant effects induced by IONPs in our experiments can only be speculated upon. Based on the literature available to date, it can be assumed that IONPs can enhance platelet aggregation. Direct addition of IONPs to mice has been shown to induced platelet aggregation in a dose-dependent manner.^59^ Two mechanisms have been proposed: first, IONPs enhance platelet aggregation by activating the GPIIb/IIIa receptor; second, they bridge adjacent non-activated platelets, thereby promoting agonist-induced aggregation.^65^ Moreover, IONPs have been shown to enhance platelet activation, as evidenced by significant increases in the soluble platelet markers sCD62P, PF4, and NAP-2 in whole blood following incubation with endothelial cells.^66^

The drastic reduction in CT observed in our experiments cannot be solely attributed to enhanced platelet function and aggregation. It is likely that IONPs also interact with coagulation factors and inhibitors. However, our ROTEM analysis does not provide sufficient resolution to determine the specific factors or inhibitors involved in these interactions. Nevertheless, evidence from the literature supports this possibility. For instance, Kottana *et al.* reported that IONPs interact with fibrinogen, inducing conformational changes in this key plasma protein.^67^ Moreover, the markedly shortened CT observed in our study aligns well with the findings of Nemmar *et al.*, who demonstrated that IONPs induce a reduction in both activated partial thromboplastin time (APTT) and prothrombin time (PT).^59^ These results further support the hypothesis that IONPs may interact directly with components of the coagulation cascade. Additionally, there is growing evidence that nanoparticles can influence coagulation inhibitors. Notably, Nemmar *et al.* reported elevated levels of plasminogen activator inhibitor-1 (PAI-1) in mice following exposure to IONPs, suggesting another potential prothrombotic mechanism.^59^ These findings highlight the urgent need for further research specifically investigating the interactions between nanoparticles and both coagulation factors and inhibitors.

It has been reported that IONPs influence blood coagulation depending on their size and surface chemistry.^65^ Interestingly, CT showed no significant correlation with the hydrodynamic diameter of the particles (0.169, *p* = 0.331, Tables S1 and S5), indicating that the overall coagulation initiation process is not directly influenced by the hydrodynamic diameter of IONPs (Fig. 3B) but rather by their mere presence in the blood samples. However, the RSM model revealed a significant, albeit subclinical, effect of synthesis conditions on CT (Table 3), while the hydrodynamic diameter alone did not predict clotting behaviour (Fig. 3B). This implies that surface chemistry or nanoparticle reactivity, rather than physical dimensions, may play a more critical role in modulating coagulation initiation.

**Table 3:** Investigated synthesis factors significantly affecting the coagulation time (CT) of the synthesised IONP. Full table in SI

Factor	Coefficient	Std. error	*t*-Statistic	*p*-Value
Fe^3+^	3.85	1.038	3.709	0.014
NaOH	−2.34	0.88	−2.665	0.045
*I* (NaOH**2)	−9.79	2.895	−3.383	0.02
Fe^2+^ : Fe^3+^	−4.72	1.382	−3.416	0.019
Fe^2+^ : NaOH	8.71	2.448	3.557	0.016
Fe^3+^ : NaOH	−4.35	1.014	−4.293	0.008

Table 3 summarises the synthesis factors that significantly affect CT, as identified by the RSM model. Fe^3+^ concentration exhibited a positive effect on CT (*β* = 3.85, *p* = 0.014, Tables 2 and 3), indicating that a 1 mM increase in Fe^3+^ concentration during synthesis results in an average prolongation of CT by 3.85 s (Fig. 3C). This effect may be attributed to altered surface oxidation states, where higher Fe^3+^ availability could lead to nanoparticles with a different Fe^2+^/Fe^3+^ ratio, potentially reducing their procoagulant activity. There is only very limited data on the interaction between IONPs and CT, as most studies focused on the effects of free iron ions. Previously, it was suggested that Fe^3+^ can prolong the clotting time by interacting with coagulation proteins.^68^ Conversely, it was also reported that Fe^3+^-modified poly(l-lactic acid) led to a decrease in CT.^69^ Additionally, it must be taken into consideration that the behaviour of IONPs, where the iron ions are complexed, cannot be directly compared to the effect of free Fe^3+^. Thus, the observed effect is likely due to changes in the surface chemistry of the particles which can impact the CT.^26,70^ These results suggest that Fe^3+^-rich surfaces potentially interact more efficiently with proteins involved in the coagulation cascade, leading to slower clot initiation.

Likewise, the ratio between Fe^3+^ and NaOH (*β* = −4.35, *p* = 0.008) negatively impacted CT (Table 3), though the interaction between both concentrations appears to be nonlinear and more complex according to the RSM model (Fig. 3D). Overall, a higher Fe^3+^ concentration relative to NaOH was associated with a prolonged CT, suggesting that excess Fe^3+^ may interfere with coagulation factor interactions or alter the surface chemistry of the IONPs, reducing their procoagulant activity. However, this effect was gradually diminished as NaOH concentrations exceeded 0.5 M, leading to a progressive acceleration of CT. This behaviour may be linked to NaOH-induced changes in nanoparticle surface chemistry, particularly through increased hydroxylation and Fe^3+^ hydrolysis, which could impact how IONPs interact with clotting factors. Supporting this, the concentration of NaOH (*β* = −9.79, *p* = 0.020) exhibited a quadratic effect on CT (Table 3), suggesting that beyond a certain threshold, excessive NaOH may disrupt the balance of nanoparticle surface properties (Fig. 3E). This could possibly be due to increased surface hydroxylation or changes in the surface chemistry, which could also be observed in the zeta potential measurements (Table S4). These changes may enhance interactions with clotting factors such as fibrinogen or prothrombin, thereby reducing their catalytic activity in clot formation.^71,72^

The observed negative correlation between the Fe^2+^/Fe^3+^ ratio in the RSM and CT (−4.72, *p* = 0.019) suggests that increasing the Fe^2+^ content by 1 mM in the synthesis accelerates coagulation by almost 5 s. Likewise, as already discussed earlier, an increased Fe^3+^ concentration leads to a prolonged CT and low concentrations of both Fe^2+^ and Fe^3+^ lead to an accelerated CT (Fig. 3F). Similarly, interactions between Fe^2+^ and NaOH (*β* = 8.71, *p* = 0.016) influenced the CT (Table 3), implying that higher Fe^2+^ compared to NaOH leads to a decreased coagulation time (Fig. 3G). This phenomenon may be attributed to several factors. First, Fe^2+^ is more chemically reactive than Fe^3+^ due to its oxidation state, potentially enhancing the surface reactivity of IONPs, thereby promoting faster interactions with factors involved in coagulation,^59^ such as fibrinogen and prothrombin. Additionally, Fe^2+^ is known for its redox activity, which may lead to the generation of ROS,^73^ potentially further influencing the coagulation cascade by activating platelet aggregation and fibrin formation.^74^

Taken together, these findings demonstrate that the synthesis conditions of IONPs can significantly influence CT, likely through alterations in nanoparticle surface chemistry and reactivity. While Fe^3+^-rich surfaces appear to prolong CT (Fig. 3C, D, and F), increasing Fe^2+^ levels were associated with faster clot initiation (Fig. 3F and G), possibly due to enhanced interactions with coagulation proteins and redox-driven activation of clotting pathways. Additionally, variations in NaOH concentrations suggest a complex interplay between surface hydroxylation and coagulation factor interactions (Fig. 3D and E), further modulating clotting dynamics. Given the limited data on IONPs in coagulation studies, these results highlight the need for further investigation into surface-specific interactions and their implications for biomedical applications.

**Fig. 4:**
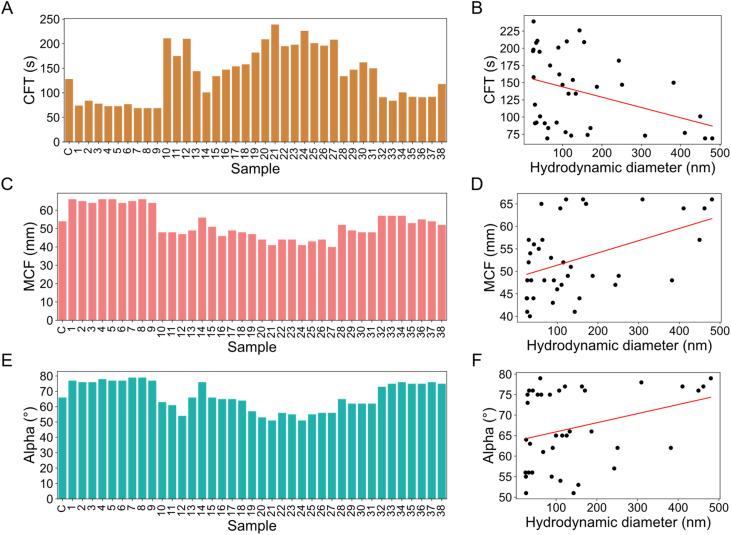
Effect of IONP synthesis parameters and hydrodynamic diameter on blood coagulation. (A, C, and E) Data for each coagulation parameter are shown alongside their (B, D, and F) respective Spearman correlations with IONP hydrodynamic diameter and corresponding regression lines (red). (A and B) Clot formation time (CFT), (C and D) mean clot firmness (MCF), and (E and F) alpha angle.

Interestingly, the IONP-induced changes of the three remaining ROTEM values, namely CFT, MCF and alpha angle, could not be explained by the RSM model (Table S6), which includes the different synthesis parameters (Table 1). Despite the high *R*^2^ values for CFT (*R*^2^ = 0.954, adjusted *R*^2^ = 0.655), the model was not statistically significant (*F*-statistic = 0.134), suggesting that synthesis parameters alone do not fully account for variations in CFT (Fig. 4A, Table S6). However, IONPs-induced changes were significantly influenced by the hydrodynamic diameter of the particles (Table S1), as indicated by a moderate negative correlation between CFT and diameter (−0.377, *p* = 0.020, Fig. 4B, Table S5). Thus, CFTs were shortened more as nanoparticle size increased. Correspondingly, larger nanoparticles were associated with a more stable fibrin network, reflected by higher MCF (Fig. 4C and D, Table S5) and alpha angle values (Fig. 4E and F, Table S5). These findings are in good agreement with previous studies which reported an accelerated fibrin polymerisation caused by IONPS obtained by ultrasound-assisted plasma discharge synthesis, leading to denser and more complex gel structures.^75,76^ These particles exhibited enhanced catalytic and enzymatic interactions compared to inert IONPs synthesised by coprecipitation.^77^

Overall, these findings highlight the distinct influence of particle size and synthesis parameters on different aspects of coagulation dynamics. While CT is not directly affected by particle size and is better explained by synthesis conditions, CFT, MCF, and alpha angle show a clear dependence on particle size, with smaller IONPs accelerating clot formation. However, the stability of MCF and alpha angle suggests that despite these changes in clot formation dynamics, the final clot structure and firmness remain intact, comparable to the control. This indicates that while IONPs influence the early stages of coagulation, they do not compromise overall clot stability, highlighting their potential for biomedical applications that require controlled coagulation interactions.

## Conclusions

This study demonstrates how varying parameters in the coprecipitation synthesis of IONPs influence their physicochemical and biological properties, providing valuable insights into their potential biomedical applications.

The regression model explaining particle size variations had an *R*^2^ of 0.91, indicating strong predictive power of the tested synthesis parameters. Although the adjusted *R*^2^ (0.74) is slightly lower than the *R*^2^, it still indicates a robust and reliable model with minimal risk of overfitting. The size of the synthesised nanoparticles was positively influenced by Fe^2+^ concentration (*β* = 1.72), the Fe^2+^/Fe^3+^ ratio (*β* = 1.02), NaOH–stirring speed interaction (*β* = 1.65), and temperature–stirring speed dynamics (*β* = 1.66). In contrast, particle size was negatively affected by stirring speed (*β* = −1.17), the Fe^2+^/NaOH ratio (*β* = −1.94), and dosing rate (*β* = −2.64). Despite substantial variation in synthesis factors, all particles remained in the magnetite phase and within the expected size range for coprecipitated IONPs (3–16 nm), underscoring the robustness of the coprecipitation method. Moreover, the ability to fine-tune size supports application-specific design, which is crucial for optimising performance in various biomedical fields.^3^

Cytotoxicity assays using 3T3 fibroblasts and HEK cells showed cell viability above 99% compared to the control across all samples. No significant correlation was observed between particle size and cell viability, supporting the conclusion that particle size variation due to synthesis conditions does not compromise biocompatibility.

In terms of blood coagulation effects, IONPs significantly reduced CTs for all tested nanoparticles by an average of 83 s, suggesting procoagulant activity. Variations in CT between the particles could be explained by the differing synthesis conditions, though only in a subclinical range. Higher Fe^3+^ concentrations prolonged CT, while elevated Fe^2+^ shortened it. NaOH concentration and Fe^2+^/Fe^3+^ ratios also modulated CT, likely *via* changes in surface chemistry and reactivity.^26,70–72^ Thus, for applications requiring minimal coagulation interaction, lower Fe^2+^ levels and post-synthesis surface modifications may be advisable. Conversely, procoagulant IONPs for haemostatic applications may benefit from higher Fe^2+^ concentrations and smaller particle sizes.

In contrast, CFT, MCF, and the alpha angle were more strongly influenced by particle size, with smaller particles accelerating clot formation but not compromising final clot firmness. Notably, the consistent clot firmness (MCF, alpha angle) despite changes in CT suggests that IONPs modulate early haemostasis without impairing final clot structure, a finding that remains underreported in current literature.

Overall, these results highlight the importance of precise control over synthesis parameters to tailor IONP properties for specific biomedical uses. While the synthesis method proved robust in producing biocompatible IONPs, the procoagulant effects highlight the need for surface modifications with citrate or antibodies to mitigate thrombosis risk.^27,78^ Future studies should explore long-term *in vivo* safety, immune interactions, and the effects of surface functionalisation on coagulation behaviour to fully establish the clinical potential of these IONPs.

## Author contributions

Conceptualisation – S. P. S.; data curation – G. C., M. R., S. P. S., V. Z.; formal analysis – M. R.; funding acquisition – S. P. S.; investigation – G. V., M. R., S. P. S., V. Z.; methodology – G. V., M. R., S. P. S., V. Z.; project administration – S. P. S.; supervision – G. V., S. P. S.; validation – G. V., M. R., S. P. S., V. Z.; visualisation – M. R.; writing – original draft – M. R.; writing – review & editing – G. V., M. R., S. P. S., V. Z.

## Conflicts of interest

There are no conflicts to declare.

## Supplementary Material

NA-007-D5NA00632E-s001

NA-007-D5NA00632E-s002

## Data Availability

The data supporting this article have been included as part of the supplementary information (SI). Supplementary information is available. See DOI: https://doi.org/10.1039/d5na00632e.
