# Supplementary material for: Influence of coprecipitation synthesis parameters on the physicochemical properties and biological effects of iron oxide nanoparticles
Source: Nanoscale Adv. 2025 Oct 2;7(22):7395–407. doi: 10.1039/d5na00632e (PMC12509087; doi:10.1039/d5na00632e)
Supplement: NA-007-D5NA00632E-s001 [file NA-007-D5NA00632E-s001.pdf]

Supplementary Information (SI) for Nanoscale Advances.
This journal is © The Royal Society of Chemistry 2025

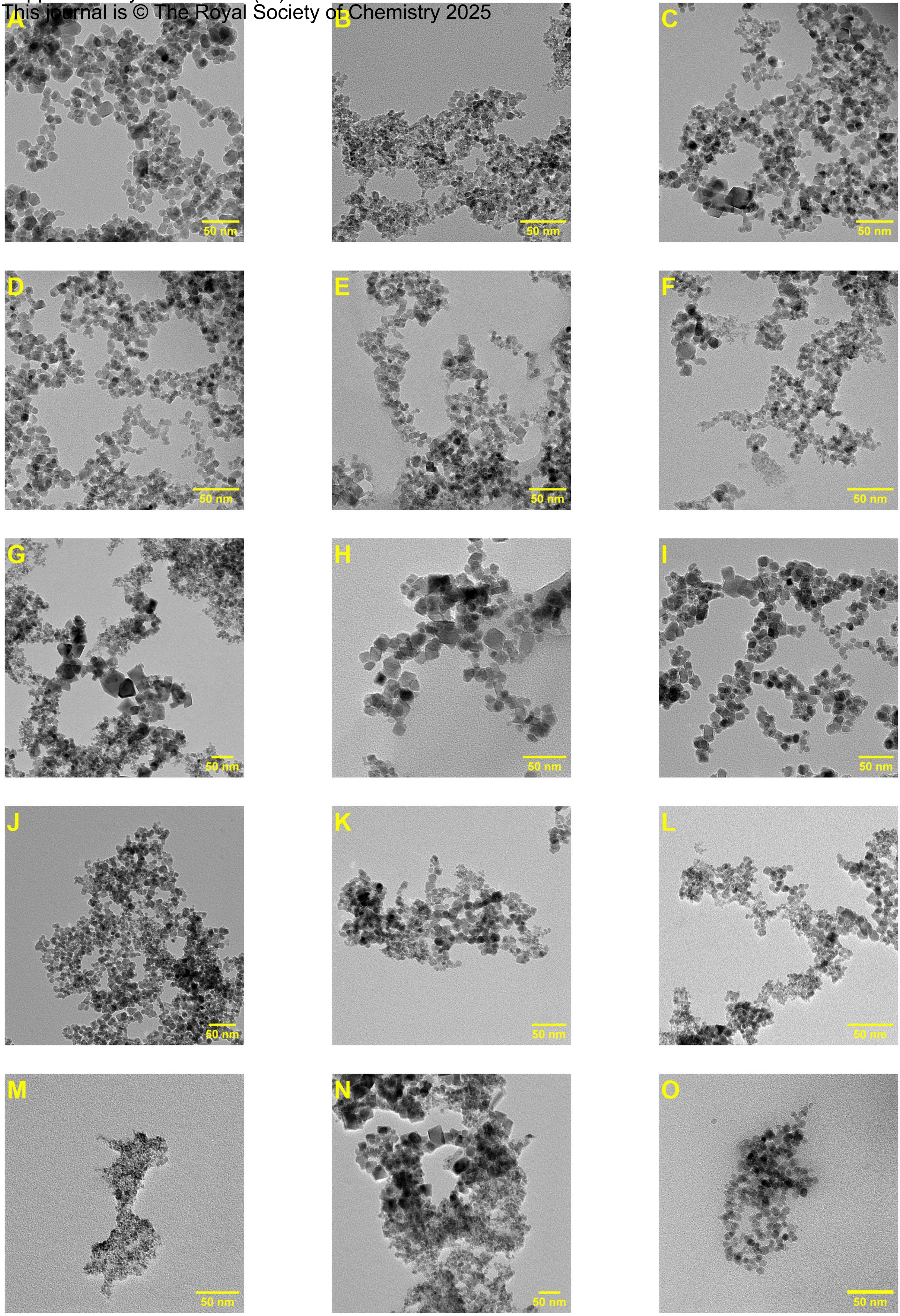

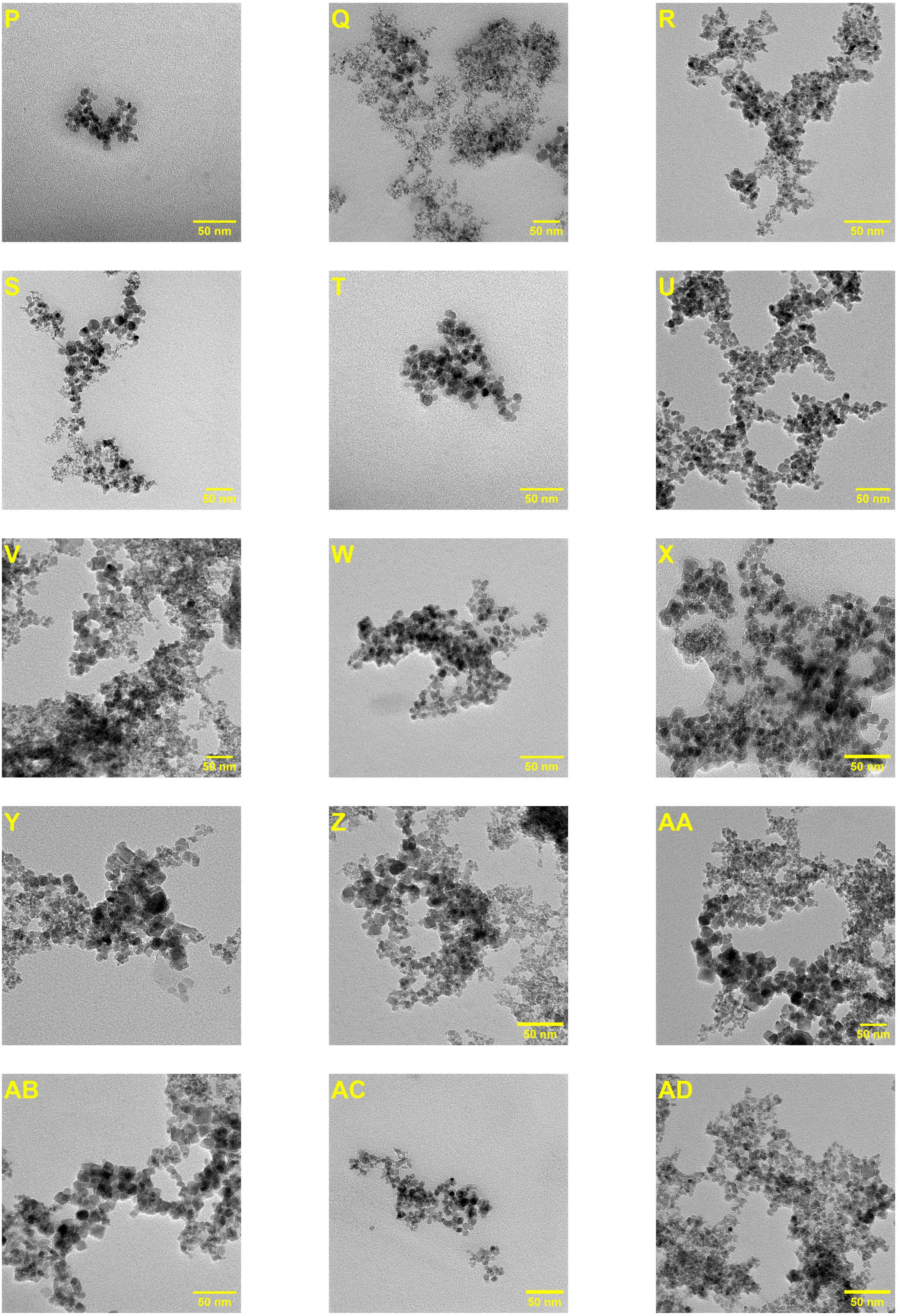
P
50 nm
Q
50 nm
R
50 nm
S
50 nm
T
50 nm
U
50 nm
V
50 nm
W
50 nm
X
50 nm
Y
Z
50 nm
AA
50 nm
AB
50 nm
AC
50 nm
AD
50 nm

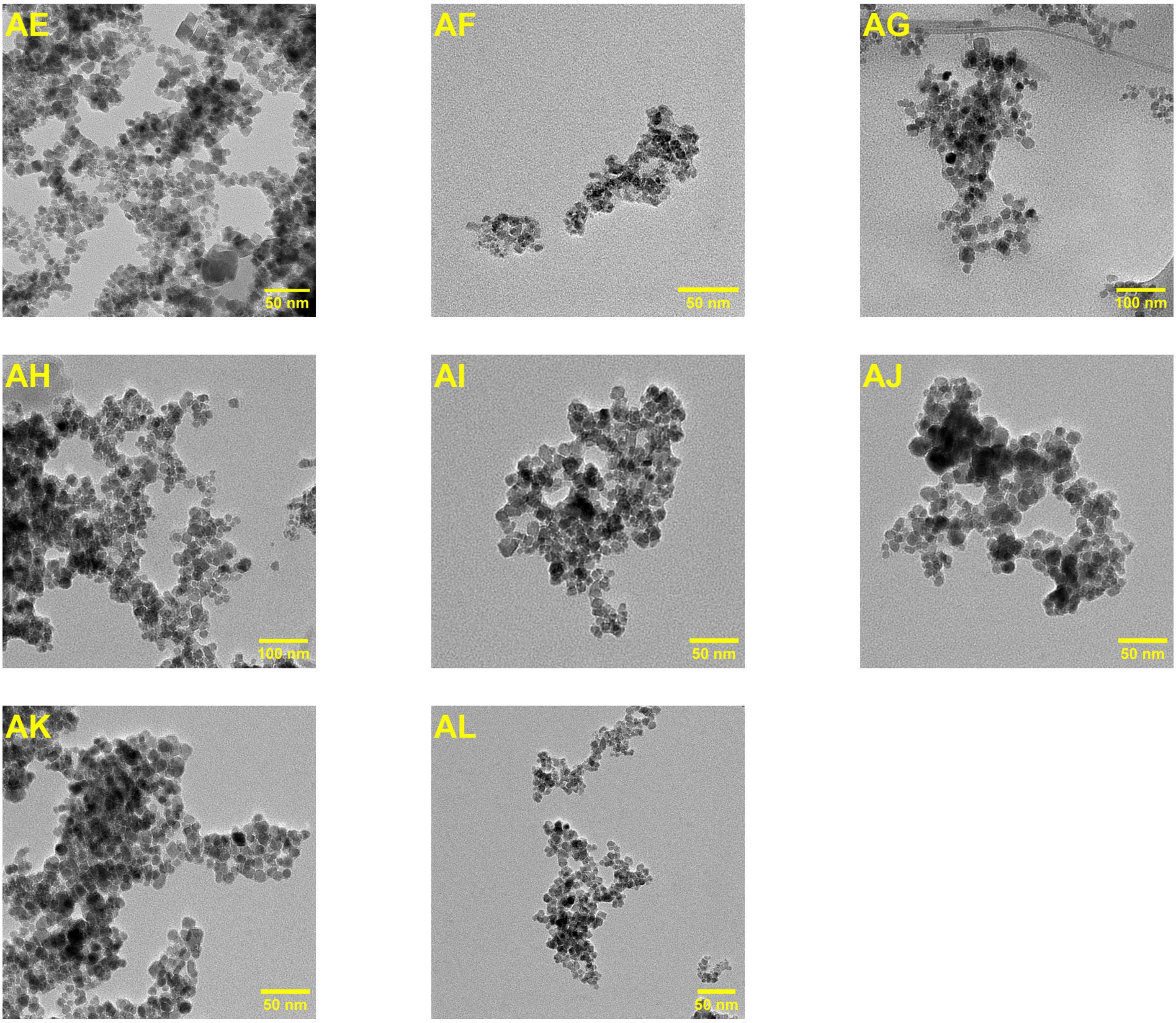

Fig S1. Representative transmission electron micrographs (TEM) of differently synthesized iron oxide nanoparticles.
