# Supplementary material for: Influence of coprecipitation synthesis parameters on the physicochemical properties and biological effects of iron oxide nanoparticles
Source: Nanoscale Adv. 2025 Oct 2;7(22):7395–407. doi: 10.1039/d5na00632e (PMC12509087; doi:10.1039/d5na00632e)
Supplement: NA-007-D5NA00632E-s002 [file NA-007-D5NA00632E-s002.pdf]

Supplementary Information (SI) for Nanoscale Advances.
This journal is © The Royal Society of Chemistry 2025

Table S1. Summary of data from transmission electron micrographs (diameter), dynamic light scattering (hydrodynamic diameter) and rotational thromoelastometry (ROTEM) analysis.

Sample	TEM Image (Fig. S1)	Diameter (nm)	Hydrodynamic diameter (mm)	CT (s)	CFT (s)	MCF (mm)	Alpha (°)
C (water)		—	163	168	128	54	66
1	A	11.2	171	87	74	66	77
2	B	5.6	107	82	84	65	76
3	C	8.4	309	86	78	64	76
4	D	9	122	78	73	66	78
5	E	8.7	410	83	73	66	77
6	F	7.3	61	84	77	64	77
7	G	7.6	480	71	69	65	79
8	H	10.1	462	81	69	66	79
9	I	9.2	36	86	69	64	77
10	J	8.1	68	76	211	48	63
11	K	7.5	110	88	175	48	61
12	L	6.1	187	85	210	47	54
13	M	3.6	43	75	144	49	66
14	N	5.1	133	80	101	56	76
15	O	6.7	100	84	134	51	66
16	P	6.6	126	92	147	46	65
17	Q	4	26	81	154	49	65
18	R	5.5	243	88	158	48	64
19	S	7	155	89	182	47	57
20	T	7.2	26	93	209	44	53
21	U	6.9	41	101	239	41	51
22	V	5.3	26	82	195	44	56
23	W	5.7	143	83	198	44	55
24	X	6.7	89	81	226	41	51
25	Y	6.3	25	78	201	43	55
26	Z	5.5	33	81	196	44	56
27	AA	5.3	115	93	208	40	56
28	AB	6	251	91	134	52	65
29	AC	5.8	92	100	147	49	62
30	AD	4.9	382	84	162	48	62
31	AE	6.2	30	106	150	48	62
32	AF	7	63	80	91	57	73
33	AG	15.5	450	91	84	57	75
34	AH	11.1	85	89	101	57	76
35	AI	8.9	54	79	92	53	75
36	AJ	9.9	33	82	91	55	75
37	AK	8.6	30	85	92	54	76
38	AL	7.1	163	75	118	52	75

Table S2. Statistical analysis of the effect of investigated synthesis factors on the particle diameter.

**R^2**	0.947			
**Adjusted R^2**	0.745			
**P(F statistics)**	0.0209			
**Factor**	**Coefficient**	**Std. Error**	**t-Statistic**	**p-Value**
Fe^{2+}	2.3374	1.043	2.241	0.075
Fe^{3+}	-0.5191	0.798	-0.65	0.544
NaOH	-0.6904	0.721	-0.957	0.382
T	-1.1651	0.779	-1.496	0.195
RPM	-1.6137	0.976	-1.653	0.159
Dosing	0.0764	0.878	0.087	0.934
I(Fe^{2+}**2)	-1.8633	0.718	-2.594	0.049
I(Fe^{3+}**2)	-0.756	1.082	-0.699	0.516
I(NaOH**2)	0.1784	1.086	0.164	0.876
I(T**2)	-0.4351	0.838	-0.519	0.626
I(RPM**2)	-1.1526	1.964	-0.587	0.583
I(Dosing**2)	-4.5203	1.632	-2.769	0.039
Fe^{2+}:Fe^{3+}	0.4159	0.946	0.44	0.679
Fe2:NaOH	-3.0447	0.985	-3.091	0.027
Fe^{2+}:T	-0.3446	1.776	-0.194	0.854
Fe^{2+}:RPM	1.2161	1.374	0.885	0.417
Fe^{2+}:Dosing	-1.2196	0.833	-1.465	0.203
Fe^{3+}:NaOH	-0.8654	0.533	-1.622	0.166
Fe^{3+}:T	-0.7519	0.729	-1.031	0.35
Fe^{3+}:RPM	-0.2603	1.455	-0.179	0.865
Fe^{3+}:Dosing	-0.1381	0.521	-0.265	0.801
NaOH:T	0.6093	0.82	0.743	0.491
NaOH:RPM	2.6886	1.116	2.408	0.061
NaOH:Dosing	-0.1803	0.427	-0.422	0.69
T:RPM	1.4922	1.483	1.006	0.361
T:Dosing	-0.2753	1.059	-0.26	0.805
RPM:Dosing	1.8063	1.094	1.652	0.159

Table S3. Statistical analysis of the effect of investigated synthesis factors on the coagulation time (CT).

**R^2**	0.971			
**Adjusted R^2**	0.816			
**P(F statistics)**	0.971			
**Factor**	**Coefficient**	**Std. Error**	**t-Statistic**	**p-Value**
T	2.2575	2.003	1.127	0.311
RPM	2.3364	1.662	1.406	0.219
Dosing	-0.9137	1.084	-0.843	0.438
I(Fe^{2+}**2)	0.4821	1.931	0.25	0.813
I(Fe^{3+}**2)	1.3091	1.521	0.861	0.429
I(NaOH**2)	-9.7928	2.895	-3.383	0.02
I(T**2)	-1.3964	2.197	-0.636	0.553
I(RPM**2)	7.6255	3.457	2.206	0.079
I(Dosing**2)	9.5333	4.201	2.269	0.072
Fe^{2+}:Fe^{3+}	-4.7224	1.382	-3.416	0.019
Fe2:NaOH	8.7082	2.448	3.557	0.016
Fe^{2+}:T	-3.6381	1.738	-2.094	0.09
Fe^{2+}:RPM	-6.8226	3.178	-2.147	0.085
Fe^{2+}:Dosing	2.8646	2.05	1.397	0.221
Fe^{3+}:NaOH	-4.3537	1.014	-4.293	0.008
Fe^{3+}:T	1.5924	1.075	1.481	0.199
Fe^{3+}:RPM	1.5887	1.399	1.136	0.308
Fe^{3+}:Dosing	1.6047	1.09	1.472	0.201
NaOH:T	-1.5359	1.193	-1.287	0.254
NaOH:RPM	-5.2615	2.284	-2.304	0.069
NaOH:Dosing	-0.5514	1.079	-0.511	0.631
T:RPM	2.7731	1.701	1.63	0.164
T:Dosing	3.2245	1.574	2.049	0.096
RPM:Dosing	-4.902	2.118	-2.315	0.068

Table S4. Zeta potential of differently synthesised iron oxide nanoparticles obtained at 25 °C and a concentration of 50 mg/L at pH 7.0.

**Sample**	**Zeta potential (mV)**
1	0.23
2	3.28
3	3.45
4	-2.94
5	1.02
6	1.57
7	2.97
8	-4.5
9	-5.35
10	-5.53
11	-0.73
12	0.08
13	3.73
14	0.69
15	-7.12
16	0.62
17	2.23
18	-0.03
19	-1.8
20	-2.09
21	-1.14
22	6.29
23	0.64
24	-1.31
25	1.77
26	7.43
27	-1
28	-4.9
29	1.46
30	2.37
31	-4.1
32	-0.66
33	-5.5
34	-7.86
35	-4.77
36	-2.08
37	-4.86
38	-1.22

Table S5. Spearman correlation between nanoparticle diameter and coagulation time (CT) clot formation time (CFT), mean clot firmness (MCF), and alpha angle.

	**Spearman correlation**	**p-value**
**CT**	0.169	0.311
**CFT**	-0.377	0.02
**MCF**	0.406	0.011
**Alpha angle**	0.349	0.032

Table S6. OLS regression model fit statistics of clot formation time (CFT), mean clot firmness (MCF), and alpha angle.

	**R^2**	**Adjusted R^2**	**P(F statistics)**
**CFT**	0.954	0.655	0.134
**MCF**	0.771	0.152	0.373
**Alpha angle**	0.912	0.615	0.0511
